# Evaluation of Antibacterial and Cytotoxic Properties of a Fluorinated Diamond-Like Carbon Coating for the Development of Antibacterial Medical Implants

**DOI:** 10.3390/antibiotics9080495

**Published:** 2020-08-09

**Authors:** Katsutaka Yonezawa, Masahito Kawaguchi, Ayumi Kaneuji, Toru Ichiseki, Yoshitsugu Iinuma, Kae Kawamura, Kazuhiro Shintani, Shinobu Oda, Makoto Taki, Norio Kawahara

**Affiliations:** 1 Department of Orthopedic Surgery, Kanazawa Medical University, 1-1 Daigaku, Uchinada-machi, Kahokugun, Ishikawa 920-0293, Japan; kats-yon@kanazawa-med.ac.jp (K.Y.); kaneuji@kanazawa-med.ac.jp (A.K.); tsy-ichi@kanazawa-med.ac.jp (T.I.); kawa@kanazawa-med.ac.jp (N.K.); 2 Department of Infectious Diseases, Kanazawa Medical University, 1-1 Daigaku, Uchinada-machi, Kahokugun, Ishikawa 920-0293, Japan; yiinuma@kanazawa-med.ac.jp; 3 Clinical Laboratory, Kanazawa Medical University Hospital, 1-1 Daigaku, Uchinada-machi, Kahokugun, Ishikawa 920-0293, Japan; kae@kanazawa-med.ac.jp; 4 Department of Mechanical Engineering, Kanazawa Institute of Technology, 7-1 Ohgigaoka, Nonoichi, Ishikawa 921-8501, Japan; shintani@neptune.kanazawa-it.ac.jp (K.S.); odas@neptune.kanazawa-it.ac.jp (S.O.); 5 Department of Technology Research, Onward Giken Corporation, Wa-13 Yoshiwara, Nomi, Ishikawa 929-0111, Japan; taki@onwardgiken.jp

**Keywords:** fluorine, diamond-like carbon, antibacterial, coating, implant, infection

## Abstract

Peri-implant infection is a serious complication in surgical procedures involving implants. We conducted an in vitro study to determine whether the use of a fluorinated diamond-like carbon (F-DLC) coating on a titanium alloy surface can prevent peri-implant infection. After applying the F-DLC, we evaluated its antibacterial and cytotoxic properties. The coating groups, containing controlled fluorine concentrations of 5.44%, 17.43%, 24.09%, and 30%, were examined for the presence of *Staphylococcus aureus* and *Escherichia coli* according to ISO 22196 for the measurement of antibacterial activity on plastics and other nonporous surfaces. Biological toxicity was evaluated using Chinese hamster V79 cells according to ISO 10993-5 for the biological evaluation of medical devices. In the control group, populations of *S. aureus* and *E. coli* substantially increased from 2.4 × 10^4^ to (1.45 ± 1.11) × 10^6^ colony-forming units (CFUs) and from 2.54 × 10^4^ to (4.04 ± 0.44) × 10^6^ CFUs, respectively. However, no bacteria colonies were detected in any F-DLC group with a fluorine concentration of ≥ 17.43%. In the biological toxicity study, an F-DLC coating with a fluorine concentration of 30% showed a colony formation rate of 105.8 ± 24.1%, which did not differ significantly from the colony formation rate of 107.5 ± 31.1% in the nontoxic control group. An F-DLC coating on titanium alloy discs showed excellent in vitro antibacterial activity with no biological toxicity.

## 1. Introduction

In orthopedic surgery, implants, such as artificial joints and internal fixation materials, are routinely used in many operations, and postoperative peri-implant infections are the most serious complications. The incidence of postoperative bone and joint infections has been reported to be 0.6%–11.9% for spine surgery [1,2,3] and 0.2%–3.8% for primary arthroplasty [4,5,6,7,8,9,10,11,12,13], and even higher incidence rates, such as 0.5%–17.3%, have been reported for revision arthroplasty [4,10,12]. Once infections occur, they are often difficult to treat because bacteria form a biofilm on the implant surface and demonstrate resistance to antibiotics [14,15,16]. Therefore, treatment is time-consuming and costly, and the patient’s quality of life substantially deteriorates [17,18,19]. Methods to impart antibacterial properties to implants themselves have been studied for use in preventing peri-implant infections. The use of various coatings on implants’ metal surfaces has been reported, including antibiotics such as gentamycin and vancomycin [20,21]; hydroxyapatite (HA) containing chlorhexidine, gentamycin, and tobramycin [22,23,24,25]; titanium dioxide, silver, and gold either applied directly or within HA containing them [26,27,28,29,30]; and iodine [31]. Some of these are already used clinically [32].

In the study we report here, we focused on a fluorinated diamond-like carbon (F-DLC) coating. Fluoride has been known for a long time to be relatively biologically safe and exert an antibacterial effect [33].

“Diamond-like carbon” is the generic name for carbon films that have diamond-like properties, such as high levels of hardness, electrical insulation, and infrared transparency [34]. F-DLC coating, which adds fluorine to the mix, is expected to be applied to implants retained in a blood vessel (e.g., vascular prosthesis, indwelling intravascular stent) because it has an anticoagulant effect [35]. Some antibacterial tests of F-DLC-coated metals and films have already been reported. However, the majority of those studies evaluated only the attachment of bacteria to the metal surface, focusing on water repellency and non-adhesiveness imparted by F-DLC, and no previous studies have been reported showing that F-DLC coating imparts infection prevention for medical implants. The culture time in those studies was as short as 3 h and, therefore, it was considered difficult to evaluate the infection protection for bioimplants [36,37,38,39]. We tried to impart an antibacterial property to implants themselves for the purpose of preventing peri-implant infections.

When using F-DLC for medical implants, it is necessary to consider not only antibacterial properties but also safety and proper concentration. The purpose of this study is to investigate whether the F-DLC coating applied to titanium shows no cytotoxicity as well as an antibacterial property. Furthermore, we investigated the proper concentration of fluorine to avoid cytotoxicity while maintaining antibacterial action.

## 2. Materials and Methods

### 2.1. Fluorinated Diamond-Like Carbon Coating

The titanium alloy Ti-6Al-4V was used as the substrate for coatings of DLC and of F-DLC. Ti-6Al-4V contains 6% aluminum and 4% vanadium and is a highly biocompatible material that is most commonly used for implants for bone fixation. The ASTM International standard was B348 grade 5 [40].

**Figure 1:**
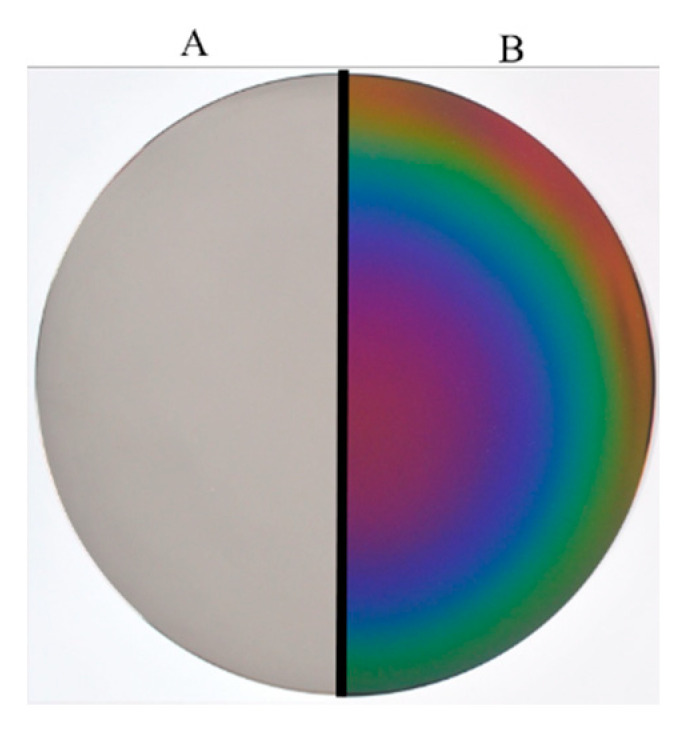
A titanium alloy disc: (**A**) before any processing; (**B**) after coating with fluorinated diamond-like carbon.

**Figure 2:**
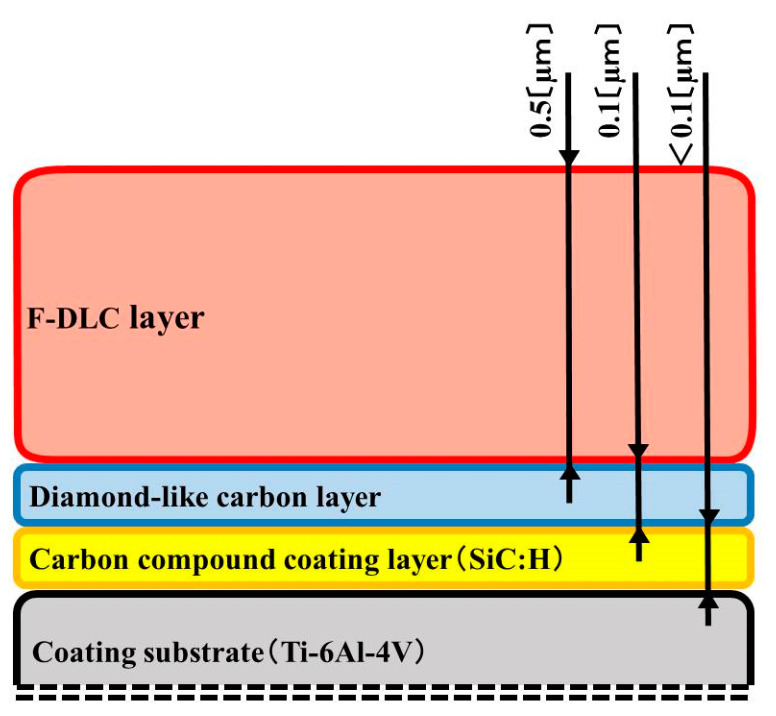
The layers of fluorinated diamond-like carbon (F-DLC) coating. First layer: SiC:H film <0.1 µm thick. Second layer: DLC film 0.1 µm thick. Third layer: F-DLC film 0.5 µm thick.

**Figure 3:**
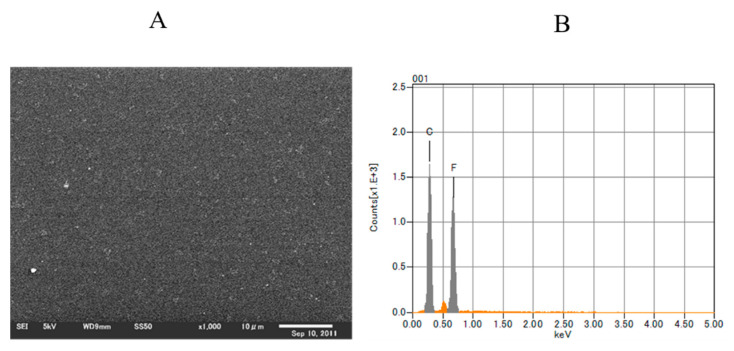
Element analysis using energy-dispersive X-ray spectroscopy (EDS) of fluorinated diamond-like carbon (F-DLC) coating of 30% fluorine: (**A**) A surface image of F-DLC coating obtained by electron microscopy (acceleration: 1000×; voltage: 5.00 kV). (**B**) EDS analysis showed that the carbon-to-fluorine ratio was 7:3. The surface fluorine concentration was 30%.

**Figure 4:**
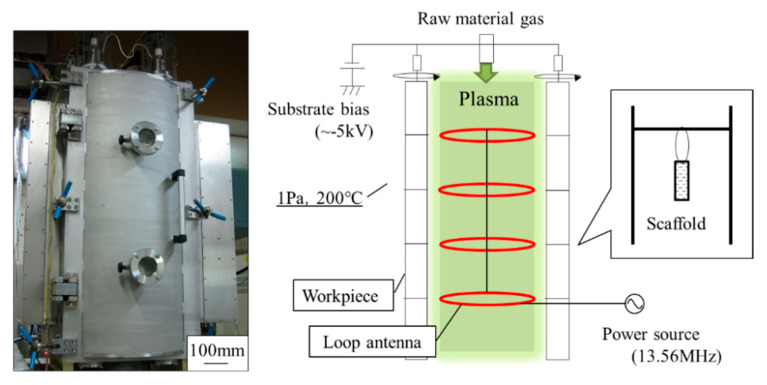
Inductively-coupled plasma device of the DLC coating device (Onward Giken, patent 4151000, 2008).

Titanium alloy discs (Semicon, Tokyo, Japan) were polished to a mean coating-surface roughness of 0.01 µm before being subjected to coating. To prevent F-DLC from coming off the metal, we etched the surface with ionic argon. We then used plasma chemical vapor deposition to apply the F-DLC [41] in three layers: (1) an SiC:H film layer of <0.1 µm, at a pressure of 0.3 Pa, using tetramethylsilane as a raw material; (2) a DLC film layer of 0.1 µm, at a pressure of 0.4 Pa, using acetylene (C2H2); and (3) an F-DLC film layer of 0.5 µm, at a pressure of 3.7 Pa, using a gaseous mixture of octafluoropropane (C3F8) and acetylene at specified ratios (Figure 1 and Figure 2). The fluorine content of the F-DLC coating was at as high a level as is currently reachable—30% (elemental fluorine content ratio: F/[C + F]; Figure 3). Plasma chemical vapor deposition to apply the F-DLC was used Inductively coupled plasma device. This equipment uses C_2_H_2_ and C_3_F_8_ as the source gas. Plasma is generated by applying a high frequency to the loop antennas arranged in parallel. A film can be formed by irradiating the sample surface with ions or radicals (Figure 4). The test pieces were autoclaved before being used for experiments.

### 2.2. Evaluation of Antibacterial Activity

The antibacterial performance of F-DLC–coated implants was evaluated in vitro using ISO standard 22196 (International Organization for Standardization) [42], the standard defining the methods for antibacterial testing of products that have undergone antimicrobial processing. The culture temperature used was 37 °C, to make the test conditions closer to those of an in vivo environment.

The bacteria used were *Staphylococcus aureus* (ATCC 29214) and *Escherichia coli* (ATCC 25922).

The test pieces were titanium-alloy discs with a 25 mm diameter and a 5 mm height, with the surface of each one polished to the same roughness. We prepared seven F-DLC–coated discs and, also, as controls, seven non-coated titanium alloy discs.

#### 2.2.1. Bacterial Fluid Preparation

Nutrient agar media were inoculated with test bacteria once a day for two days before the activated bacteria were dissolved in 1/500-diluted nutrient broth (NSU05511, Nissui Pharmaceutical Co., Ltd., Tokyo, Japan) to prepare bacterial solutions of 3.0 × 105 cells/mL, 0.2 mL each of which was dropped onto test samples. Then the test samples were covered with alcohol-sterilized, 20-mm-diameter polyethylene sheets (IC-3-2454-03, As One Corp., Osaka, Japan) and were cultured in a 37 °C incubator at 90% relative humidity for 24 h. Then the test pieces and polyethylene sheets were washed with 10 mL each of soybean-casein digest broth with lecithin and polysorbate 80 medium (WK39500265, Wako Pure Chemical Industries, Ltd., Osaka, Japan). Undiluted solution and 10-fold, 100-fold, and 1000-fold serial dilutions of the wash fluids were prepared with 1.56 × 10–4 mol/L phosphate-buffered saline, made by diluting a phosphate buffer solution (WK16819965, Wako Pure Chemical Industries, Ltd.) with physiological saline.

#### 2.2.2. Viable Cell Count and Antibacterial Activity Evaluation

Nutrient agar media (IC-2-8856-01, As One Corp.) were inoculated with 0.1 mL of undiluted solution and each dilution made by the method described earlier, and then cultured in a 37 °C incubator for 48 h before colonies were counted. The viable cell count was computed from the colony counts and dilution factors and was used to evaluate the antibacterial activity.

Using ISO 22196, we evaluated antibacterial activity with the following equation:

R = (Ut – U0) – (At – U0)(1)

where R = antibacterial activity; Ut = common logarithm of the count of viable bacteria recovered after 24 h from the sample piece without an antimicrobial coating; At = common logarithm of the count of viable bacteria recovered after 24 h from the sample piece with an antimicrobial coating; and U0 = mean common logarithm of the count of viable bacteria recovered immediately after inoculation from the sample piece without an antimicrobial coating.

The antibacterial activity showed the difference in the logarithm of the viable cell count after inoculation of bacteria between the antibacterial-treated product and the untreated product. The bacterial growth rate was ≤1/100 compared with materials with antibacterial activity of ≥2 without antibacterial treatment.

### 2.3. Evaluation of Cytotoxicity

ISO 10993-5 was used for evaluation of the biological toxicity of F-DLC-coated implants in vitro [43]. ISO 10993 is an international standard defining the biological evaluation of medical devices. The experimental method used was based on ISO 10993-5, in particular ISO 10993-5 Annex B, with which cytotoxicity can be measured quantitatively.

#### 2.3.1. Preparation of Test Pieces and Sample Extract Fluids

Test sample pieces were four F-DLC–coated discs with a content of 30% fluorine, with a 65-mm diameter and a 5 mm height; four titanium-alloy discs without any coating were used as control samples for comparison.

Sample fluid extracts were prepared by immersing each test sample in minimum essential medium (MEM) 05 medium (6 cm^2^/mL) for 24 h in a 5% CO2 incubator at 37 °C. Ingredients in the MEM05 medium were as follows: 500 mL of MEM with no glutamine (RO-10370021, Life Technologies, Tokyo, Japan), 53.5 mL of fetal bovine serum (RO-26140079, Life Technologies), 10 mL of sodium pyruvate (WK19014881, Wako Pure Chemical Industries, Ltd.), and 0.292 g of l-glutamic acid (WK07305391, Wako Pure Chemical Industries, Ltd.).

#### 2.3.2. Test Material for Cytotoxicity Evaluation

The following controls for cytotoxicity were used: 0.1% zinc diethyldithiocarbamate (ZDEC, Food and Drug Safety Center, Hatano Research Institute, Kanagawa, Japan) and 0.25% zinc dibutyldithiocarbamate (ZDBC, Food and Drug Safety Center) as positive controls, and high-density polyethylene (Food and Drug Safety Center) as negative controls. A sample extract fluid was prepared from each sample.

#### 2.3.3. Cell Culture

Chinese hamster V79 cells (DV-EC86041102, European Collection of Authenticated Cell Cultures, Wiltshire, England) were used for our cytotoxicity study. First, MEM05 medium was prepared to culture the cells. A V79 cell suspension of 33.3 cells/mL in MEM05 medium was dispensed in 3 mL portions into a well plate, and the plate was then incubated in a 5% CO2 incubator at 37 °C for 24 h. Next, the MEM05 medium in the well plate was discarded, and sample extract fluid was poured into each well plate. Two milliliters each of the undiluted extracts and of 1/2, 1/4, 1/8, 1/16, and 1/32 serial dilutions prepared from the sample extracts were dispensed in drops, and the plate was incubated in a 5% CO2 incubator at 37 °C for six days. Cell colonies formed in each well were fixed with methanol, stained with 5% Giemsa solution (prepared by diluting WK07904391 (Wako Pure Chemical Industries, Ltd.)), and counted.

Using ISO 10993-5, we computed the colony formation rate by dividing the number of colonies in each well by the number of colonies in the non-extract fluid (control). The sample was considered potentially cytotoxic when the colony formation rate at the highest concentration of the sample extract (100% extract) was <70% of the control rate; the sample was considered non-cytotoxic when the colony formation rate was ≥70% of the control rate [43].

### 2.4. Evaluation of Antibacterial Activity by Changes in Fluorine Density

**Figure 5:**
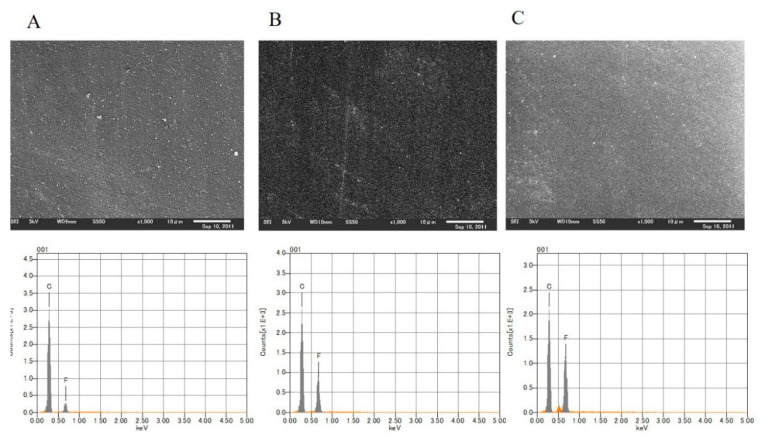
Surface images and element analysis by energy-dispersive X-ray spectroscopy of the fluorine content of the fluorinated diamond-like carbon (F-DLC) coating for various discs: (**A**) Content of 5.44%. (**B**) Content of 17.46%. (**C**) Content of 24.09%.

The antibacterial activity imparted by the F-DLC coating was evaluated by measuring the minimum inhibitory concentration against bacteria. The effective antibacterial fluorine content was determined using F-DLC–coated samples with different fluorine contents. Titanium-alloy discs with a 25 mm diameter and 5 mm height were surface-polished to prepare plates with a uniform surface roughness. The fluorine content in the coat was changed by adjusting the fluorine gas concentration used in the F-DLC coating process. As a result, F-DLC-coated test pieces with fluorine contents of 5.44%, 17.46%, and 24.09% were obtained (Figure 5). For each fluorine content, two discs were prepared and used for the antibacterial activity assay in experiment 1.

For the continuous variables of values calculated from this experiment, two groups were compared by t-test. Any *p* value of <0.05 was considered statistically significant. Values were calculated using Excel 2013 (Microsoft Corp., Redmond, VA, USA).

## 3. Results

### 3.1. Evaluation of Antibacterial Activity

**Figure 6:**
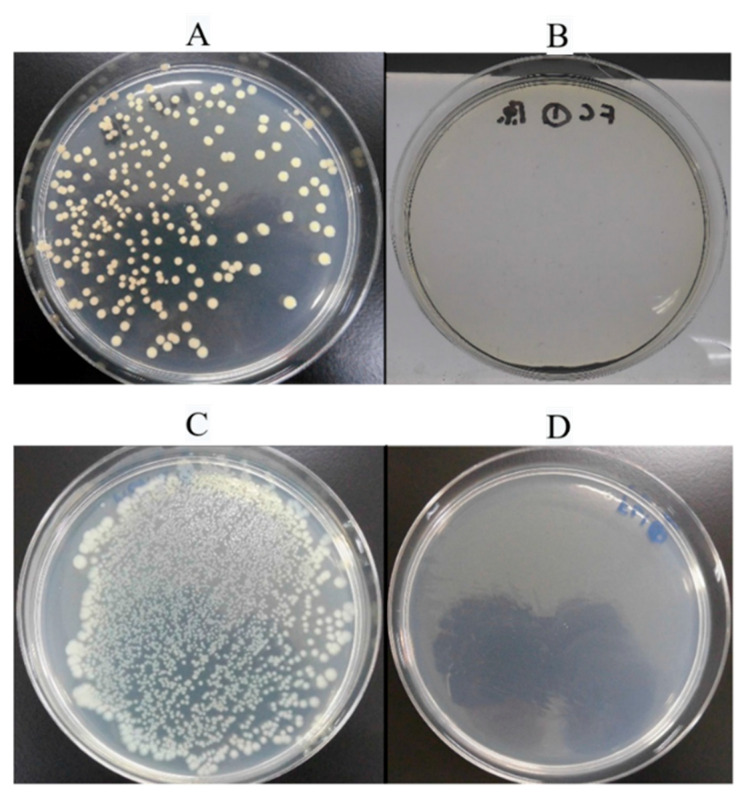
Antibacterial activity: (**A**) *Staphylococcus aureus* cultured on a noncoated titanium alloy disc. (**B**) *S. aureus* cultured on a disc with coated with fluorinated diamond-like carbon (F-DLC). (**C**) *Escherichia coli* cultured on a noncoated titanium-alloy disc. (**D**) *E. coli* cultured on a disc coated with F-DLC.

Using ISO 22109, we counted the number of bacterial colonies 24 h after culturing bacteria on noncoated and F-DLC coated discs. Seven samples were tested in each group (Table 1). For the group of noncoated discs, *S. aureus* and *E. coli* counts had increased from 2.4 × 104 colony-forming units (CFUs) and 2.54 × 104 CFUs to (1.45 ± 1.11) × 106 CFUs and (4.04 ± 0.44) × 106 CFUs, respectively (*p* < 0.005). For the group of F-DLC-coated discs with 30% fluorine, no bacteria were detected for any cultured samples, including those with media inoculated with the undiluted stock solution (Figure 6). The antibacterial activity values were 4.73 ± 0.35 and 5.30 ± 0.05 for *S. aureus* and *E. coli*, respectively, indicating the antibacterial activity of F-DLC-coated samples. The activity levels show the difference in the logarithms of the viable cell count, after inoculation with bacteria, between the antibacterial-treated product and the untreated product. Therefore, with the F-DLC coating, bacterial growth was suppressed to 1/10,000 in *S. aureus* and 1/100,000 in *E. coli*, in comparison with the noncoated group.

### 3.2. Evaluation of Cytotoxicity

**Figure 7:**
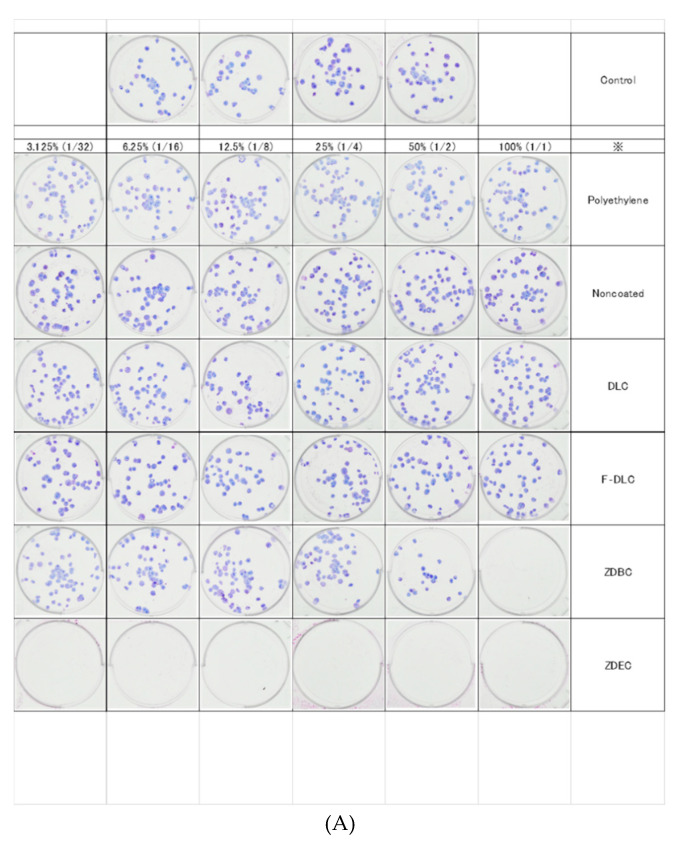

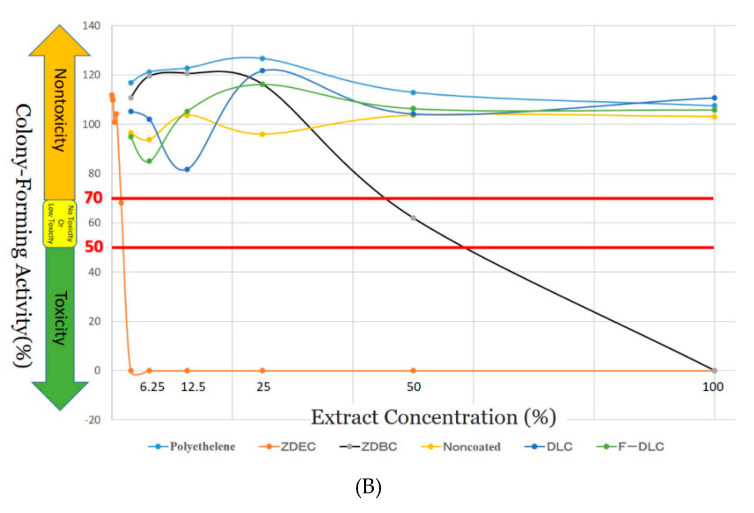
Colony-forming activity by extract concentration. (**A**) Number of viable V79 colonies after cultivation with test discs. V79 colonies in ZDBC and ZDEC were not detected in the undiluted extracts. Regarding viable V79 colonies for polyethylene and for F-DLC in titanium-alloy discs without coating, DLC was detected to the same extent in all extract dilution levels. (**B**) A graph of colony-forming activity by extract concentration. The activity level in ZDEC and ZDBC was 0% in the undiluted extracts, which shows that the sample are potentially cytotoxic. However, the activity in polyethylene and F-DLC-coated samples was >70%, which shows that the samples were likely non-cytotoxic. DLC, diamond-like carbon; F-DLC, fluorinated diamond-like carbon; ZDBC, 0.25% zinc dibutyldithiocarbamate; ZDEC, 0.1% zinc diethyldithiocarbamate.

The number of colonies and the colony formation rate for Chinese hamster V79 cells cultured for six days with sample extract solutions were determined (Table 2). The 0.1% ZDEC and 0.25% ZDBC in the toxicity group did not form colonies, and the cells were completely destroyed. Using the non-extract fluid as a control, we found that the colony formation rate for the undiluted extract of the F-DLC-coated sample (30% fluorine) was 105.8 ± 24.1%. The colony-forming activity of the polyethylene in the nontoxicity target group was 107.5 ± 31.1%, and there was no significant difference between groups (*p* = 0.94), indicating the nontoxicity of the F-DLC coating (Figure 7A,B).

### 3.3. Evaluation of Antibacterial Activity by Changes in Fluorine Density

Bacterial colony counts determined after culturing bacteria for 24 h with F-DLC-coated discs having different fluorine contents according to ISO 22109 are shown in Table 3. After incubation with *S. aureus* and *E. coli* of F-DLC-coated plates containing 17.46% and 24.09% fluorine, respectively, for 24 h, bacteria inoculations added before incubation formed no detectable bacterial colonies, which was the same as the result for F-DLC plates containing 30% fluorine. However, when F-DLC-coated plates containing 5.44% fluorine were used, *S. aureus* and *E. coli* counts increased from 4.45 × 104 CFUs and 2.54 × 104 CFUs to (1.51 ± 0.01) × 106 CFUs and (8.59 ± 0.16) × 106 CFUs, respectively, and no antibacterial activity was observed (*S. aureus*, *p* < 0.05; *E. coli*, *p* < 0.002).

From these results, effective antibacterial properties were shown when the concentration of F-DLC was ≥ 17.46%.

## 4. Discussion

Fluoride has been known for a long time to have an antibacterial effect and, thus, an appropriate amount of fluoride has been added to drinking water in North America since the 1940s to help prevent dental caries [33,44,45,46].

Fluorine inhibits some phosphoglyceromutases and enolases, which are the major enzymes involved in glycolysis, at concentrations of 0.5–1 ppm, and potently inhibits bacteria growth, hereby preventing dental caries at concentrations of 5–10 ppm [33,47]. In addition, fluorine has been reported to inhibit cellular Na+/K+-ATPase in the presence of divalent cations [48,49,50]. Moreover, fluorine is found at about a level of 0.0037% in the body, which is the second highest level, next to iron, among biological trace elements; its blood level is a mean of 17.5 ± 19.7 µg/L (in humans 15–90 years old; no gender difference) [51]. The mean daily fluorine intake in humans is 0.05 mg/kg, and fluorine has a low biological toxicity, with a reported upper intake dose level of 0.1 mg/kg/d) [52]. Furthermore, unlike what is the situation for antibiotics, there are no known cases of acquired fluorine resistance in bacteria, and its use is considered to be safe.

On the basis of on international standards, we evaluated the antibacterial effects and cytotoxicity for titanium alloy discs coated with fluorine. Testing revealed a very high antibacterial level, and we found no cytotoxicity when the fluorine concentration was ≤30%. In examining the minimum bacterial growth-inhibitory concentration of fluorine, we found no antibacterial properties at a fluorine concentration of 5.44%, and we found that neither *S. aureus* nor *E. coli* showed colony growth when the concentration was ≥17.46%. This result suggests that the antibacterial level of a titanium alloy coated with F-DLC depends on the fluorine concentration on the alloy’s surface.

Methods for imparting an antibacterial property to implants have been studied by researchers in various locations to determine their usefulness in preventing peri-implant infections. However, these studies employed different testing methods and so cannot be compared straightforwardly.

Silver-containing hydroxyapatite coating (Ag-HA coating), which is used clinically, has been reported to have antibacterial activity values of 5.0 for *S. aureus* and 4.1 for *E. coli* as measured according to ISO 22196, with a colony formation rate of 71.4% on biological toxicity tests done as specified by ISO 10993-5. In our study, the F-DLC coating had antibacterial activity values of 4.73 and 3.30 for *S. aureus* and *E. coli*, respectively, showing a colony formation rate of 105.8% on biological toxicity tests. These results indicate that an F-DLC coating and an Ag-HA coating impart comparable levels of antibacterial activity, but the former is associated with no cytotoxicity. Furthermore, some studies have been reported to try to obtain antibacterial properties by adding silver or copper to DLC. These studies demonstrated that bacteria were suppressed after cultured for 24 h [53,54]. On the other hand, in this study, no bacteria were detected after being cultured for 24 h. However, these studies performed different testing methods and so cannot be compared directly. In addition, there have been no reports of bacteria with acquired resistance to fluorine. Therefore, F-DLC may be a useful coating material for long-term implants, such as prosthetic implants. In addition, most implant materials currently used in orthopedic surgery, such as titanium alloy, cobalt chromium alloy, and stainless steel, can be coated with F-DLC.

Our study has several limitations:

- Since this was an in vitro study, it is necessary to confirm whether similar safety and antibacterial efficacy are present in vivo.
- The coating’s antibacterial effects on other bacteria, such as coagulase-negative *staphylococci* and *pneumococci*, remain to be determined.
- We did not rule out the possibility that the fluorine coating reduced the bone affinity of DLC in vivo.
- It is unknown how long fluorine can maintain its antibacterial properties in vivo.
- Although F-DLC has low cytotoxicity, it is necessary to confirm whether biotransformation of the immune system or of the metabolic system occurs.

## 5. Conclusions

An F-DLC coating on titanium alloy discs showed excellent in vitro antibacterial activity with no biological toxicity. Our findings show that an F-DLC coating may be a revolutionary new technology for preventing peri-implant infections.

## Figures and Tables

**Table 1:** Number of viable bacteria on the disc.

Material	Bacterium	Number of Viable Bacteria (CFUs)		*p*-Value
		Before Incubation	After Incubation	
Fluorinated diamond-like carbon coating	*Staphylococcus aureus*	2.4 × 10^4^	<20 (not detected)	NA
	*Escherichia coli*	2.54 × 10^4^	<20 (not detected)	NA
Noncoated titanium-alloy disc	*Staphylococcus aureus*	2.4 × 10^4^	(1.45 ± 1.11) × 10^6^	<0.001
	*Escherichia coli*	2.54 × 10^4^	(4.04 ± 0.44) × 10^6^	<0.001

Abbreviations: CFU, colony-forming unit. NA, not applicable because there were no values after incubation. When no bacteria were detected after incubation, the number of viable bacteria was less than the amount of soybean-casein digest broth with lecithin and polysorbate 80 medium. The values are given as the mean ± standard deviation.

**Table 2:** Number of cells in the V79 colonies and average colony-forming activities.

Material	Number of Cells in the V79 Colonies (CFUs)	Colony-Forming Activity (%)
Non-extract fluid	45.6 ± 11.9	100 ± 17.1
Polyethylene	49 ± 14.1	107.5 ± 31.1
0.1% zinc diethyldithiocarbamate	0 ± 0	0 ± 0
0.25% zinc dibutyldithiocarbamate	0 ± 0	0 ± 0
Titanium alloy without coating	47 ± 13.6	103.1 ± 29.8
Fluorinated diamond-like carbon coating	48.25 ± 11.0	105.8 ± 24.1

Abbreviations: CFU, colony-forming unit. Number of cells were cultured by 100% extraction medium, and the colonies of 4 samples were averaged. There were no significant differences in the level of colony-forming activity of titanium alloy and fluorinated diamond-like carbon coating in comparison with that of polyethylene (*p* = 0.86 and 0.94, respectively). The values are given as the mean ± standard deviation.

**Table 3:** Number of viable bacteria on the disc at different fluorine concentrations.

Material	Density (%)	Bacterium	Number of Viable Bacteria (CFUs)		*p*-Valuea
			Before Incubation	After Incubation	
Fluorinated diamond-like carbon coating	24.09	*S. aureus*	4.45 × 104	<20 (not detected)	NA
		*E. coli*	2.54 × 104	<20 (not detected)	NA
	17.46	*S. aureus*	4.45 × 104	<20 (not detected)	NA
		*E. coli*	2.54 × 104	<20 (not detected)	MA
	5.44	*S. aureus*	4.45 × 104	(1.51 ± 0.01) × 106	<0.05
		*E. coli*	2.54 × 104	(8.59 ± 0.16) × 106	<0.01

Abbreviations: CFU, colony-forming unit; *E. coli*, *Escherichia coli; S. aureus, Staphylococcus aureus*. a Not applicable because there were no values after incubation. The values are given as the mean ± standard deviation.
